# Same-day comparative protocol PET/CT-PET/MRI [^68^ Ga]Ga-DOTA-TOC in paragangliomas and pheochromocytomas: an approach to personalized medicine

**DOI:** 10.1186/s40644-023-00521-6

**Published:** 2023-01-10

**Authors:** Stefan Prado-Wohlwend, Mónica Ballesta-Moratalla, Irene Torres-Espallardo, María Isabel del Olmo-García, Pilar Bello-Arques, Consuelo Olivas-Arroyo, Juan Francisco Merino-Torres

**Affiliations:** 1 Department of Nuclear Medicine, University and Polytechnic Hospital La Fe, Valencia, Spain; 2 Medical Imaging Clinical Area, University and Polytechnic Hospital La Fe, Valencia, Spain; 3 Department of Radiology, University and Polytechnic Hospital La Fe, Valencia, Spain; 4 Hospital Radiophysics University and Polytechnic Hospital La Fe, Valencia, Spain; 5 Department of Endocrinology and Nutrition, University and Polytechnic Hospital La Fe, Valencia, Spain; 6 Joint Research Unit On Endocrinology, Nutrition and Clinical Dietetics, University of Valencia-Health Research Institute La Fe, Valencia, Spain; 7 Hospital Radiopharmacy, Department of Nuclear Medicine, University and Polytechnic Hospital La Fe, Valencia, Spain; 8 Medicine Department, Universitat de València, Valencia, Spain

**Keywords:** Paraganglioma, Pheochromocytoma, PET/MRI, PET/CT, [^68^ Ga]Ga-DOTA-TOC, Theragnosis, Personalized medicine

## Abstract

**Background:**

PET/MRI is an emerging imaging modality which enables the evaluation and quantification of biochemical processes in tissues, complemented with accurate anatomical information and low radiation exposure. In the framework of theragnosis, PET/MRI is of special interest due to its ability to delineate small lesions, adequately quantify them, and therefore to plan targeted therapies. The aim of this study was to validate the diagnostic performance of [^68^ Ga]Ga-DOTA-TOC PET/MRI compared to PET/CT in advanced disease paragangliomas and pheochromocytomas (PGGLs) to assess in which clinical settings, PET/MRI may have a greater diagnostic yield.

**Methods:**

We performed a same-day protocol with consecutive acquisition of a PET/CT and a PET/MRI after a single [^68^ Ga]Ga-DOTA-TOC injection in 25 patients. Intermodality agreement, Krenning Score (KS), SUVmax (Standard Uptake Value), target-to-liver-ratio (TLR), clinical setting, location, and size were assessed.

**Results:**

The diagnostic accuracy with PET/MRI increased by 14.6% compared to PET/CT especially in bone and liver locations (mean size of new lesions was 3.73 mm). PET/MRI revealed a higher overall lesion uptake than PET/CT (TLR 4.12 vs 2.44) and implied an upward elevation of the KS in up to 60% of patients. The KS changed in 30.4% of the evaluated lesions (mean size 11.89 mm), in 18.4% of the lesions it increased from KS 2 on PET/CT to a KS ≥ 3 on PET/MRI and 24.96% of the lesions per patient with multifocal disease displayed a KS ≥ 3 on PET/MR, that were not detected or showed lower KS on PET/CT. In 12% of patients, PET/MRI modified clinical management.

**Conclusions:**

PET/MRI showed minor advantages over conventional PET/CT in the detection of new lesions but increased the intensity of SSRs expression in a significant number of them, opening the door to select which patients and clinical settings can benefit from performing PET/MRI.

## Background

Paragangliomas (PGLs) and pheochromocytomas (PHEOs) are rare neuroendocrine tumors (NETs) derived from chromaffin cells of the adrenal medulla and extra-adrenal ganglia. These tumors collectively abbreviated as PGGLs, can be located from the skull to the sacrum. Most of them (80–90%) are PHEOs derived from the adrenal medulla. PGLs are less common extra-adrenal tumors derived from sympathetic or parasympathetic ganglia [1].

The annual incidence of PGGLs ranges between 1 and 2 cases per million, with a peak incidence between the third and fifth decade of life and overall survival of 63% at 5 years. PGGLs are the most heritable human tumors, with at least 30–40% of them determined by a germinal autosomal dominant mutation. 32 genes related to this disease have been identified up to now [2–5].

If locally unresectable disease or distant metastases, imaging studies should be performed at an interval of 12 weeks to 12 months. In germline mutations, the timing of the follow-up should be based on which gene is affected. As many young patients will require lifelong surveillance, MRI may be the preferable imaging modality to limit radiation exposure [6]. Among functional imaging studies, somatostatin receptor (SSRs) radiopharmaceuticals suited for PET images ([^68^ Ga]Ga-DOTA-TATE, DOTA-NOC, and DOTA-TOC), due to a high affinity for SSRs and a high spatial resolution, have an increased diagnostic accuracy over the conventional imaging and allow the quantification of the SSR expression [7, 8]. In this context, PET/MRI is an emerging imaging modality which in recent years is moving from the research area to clinical application. The combination of MRI and PET imaging modalities allows the evaluation of biochemical processes in tissues, complemented with anatomical information with high soft tissue contrast and low radiation exposure [9].

In the framework of theragnosis, it is not only important to detect the lesions, but also to adequately quantify their uptake intensity. If Peptide Receptor Radionuclide Therapy (PRRT) is considered, an adequate intensity of uptake is required with a Krenning score ≥ 3 (KS), so a better detection of the intensity in the expression of SSRs may have therapeutic implications [10]. 

Given the limited availability and cost of PET/MRI equipment and considering the lower tolerance of patients to this lengthy acquisition protocol, it is necessary to evaluate which patients will benefit from this imaging study.

The aim of this study is to validate the usefulness and diagnostic performance of [^68^ Ga]Ga-DOTA-TOC PET/MRI compared with the current standard of care [^68^ Ga]Ga-DOTA-TOC PET/CT in PGGLs, to assess the intermodality agreement between both tests, to determine in which clinical settings PET/MRI may have a greater diagnostic yield, and finally to select which patients can benefit from it.

## Material and methods

This is a prospective study enrolling consecutively patients with locally advanced or metastatic histologically proven PGGLs. The patients were referred to our NETs accredited tertiary care center (recognized by the regional administration) for staging, follow-up or therapeutic decision between September 2020 and June 2022. This study was performed with the approval of the local ethics committee. Exclusion criteria were pregnancy, age < 18 years, contraindication to MRI, and inability to understand the protocol or to give an informed consent form.

We performed a same-day protocol in 25 patients with metastatic or unresectable PGGLs. A consecutive acquisition of PET/CT and PET/MRI was completed after a single intravenous radiotracer injection of approximately 185 MBq of [^68^ Ga]Ga-DOTA-TOC with no radiological contrast. A whole-body PET/CT scan was firstly acquired, covering the anatomy from the skull to the upper thigh, followed immediately by a PET/MRI scan, covering the same anatomy. The PET/CT equipment was Gemini TF 64 (PHILIPS)®. For PET, this scanner offers a sensitivity of 6.6 cps/kBq, a transaxial resolution of 4.7 mm, and a field of view (FOV) of 18 cm, with fully 3D time-of-flight (TOF) technology [11]. In PET/CT, images are not acquired simultaneously, CT is performed first and then PET. The PET/MRI equipment was a SIGNA™ PET/MRI (GE). It has a 3 T MRI and the PET offers a sensitivity of 23.3 cps/kBq, a transaxial resolution of 3.7–4.2 mm, FOV of 25 cm, TOF, simultaneous acquisition and incorporates a Point-Spread Function (PSF) [12]. For every bed position in PET/MRI, attenuation information was collected by acquiring an 18-s LAVA-Flex scan. We acquired T1-weighted out-of-phase and in-phase sequences, T2-weighted sequences, Fat-suppressed T2-weighted sequences (FS), Short-TI Inversion Recovery, diffusion-weighted sequences (DWI) and a volumetric interpolated breath-hold examination sequence.

Nuclear medicine physicians and radiologists with expertise in the field assessed the images. A blind review of the images was performed first of the PET/CT and then of the PET/MRI. Discrepancies between reviewers were solved jointly by consensus. Intermodality agreement between radiotracer uptake, KS, SUVmax (Standard Uptake Value) and target-to-liver-ratio (TLR), to normalize lesion uptake to baseline liver activity, were assessed for all radiopharmaceutical foci and correlated to the clinical settings, location, and size of the lesions. Hepatic and splenic SUVmean were also quantified. All radiopharmaceutical uptakes were evaluated. Focal non-physiological uptakes in PET with correlation on CT or MRI were considered as tumoral lesions. Uptakes with established benign MRI or CT features and physiological uptakes with no underlying lesions on CT or MRI were rated as negative. Focal bone uptakes, not attributable to insertional or inflammatory pathology were considered malignant. Non-physiological focal uptakes in PET without underlying lesions on CT or MRI were assessed in the context of each case, especially in patients with known millimetric lesions. A region-based involvement evaluation included the spread in new anatomic regions, new lesions in regions already affected, discrepancy between both studies, and whether these findings involved a change in clinical management.

Previous and/or follow-up examinations and histological data taken from surgery or biopsy were used to confirm the lesions. SUVmax, TLR and size were also recorded to assess the intermodality agreement. SUVmax was recorded by generating isocontour volumes of interest that included all voxels greater than 40%, both in lesions and in healthy liver tissue.

Statistical studies were performed with a 95% confidence interval. TLR obtained from PET/CT and PET/MRI were compared statistically by the U test of Mann–Whitney for independent samples because they do not follow a normal distribution. Lesions were grouped according to their size by quartiles to evaluate the difference in uptake between the groups. Differences between uptake and the lesion size groups were statistically evaluated with the non-parametric Kruskall Wallis test. Differences between both imaging tests, multifocal disease and the genetic syndrome were assessed by a least squares regression. The intermodality agreement between both imaging studies was calculated by using the Kappa coefficient.

## Results

Out of 30 patients included in the study, 5 did not complete the protocol: 2 of them due to technical problems with the PET/MRI equipment, 2 of them did not tolerate the protocol, and in 1 patient with a suspected paraganglioma, it was ruled out by imaging findings and later confirmed after surgery.

Twenty-five patients completed the protocol, and 125 lesions were evaluated. A mean of 183.52 Mbq of [^68^ Ga]Ga-DOTA-TOC (165.76–203.13) was administered. The mean time from radiotracer injection to acquisition was 65 min (45–101) for PET/CT and 109 min (81–165) for PET/MRI. The activity corrected for radioactive decay at the start of the acquisition was 93.61 Mbq for PET/CT and 59.57 Mbq for PET/MRI. The characteristics of the patients are summarized in Tables 1 and 2.

**Table 1:** Overall characteristics of the study patients

Gender	Female 12
	Male 13
Age	47.52 years (20–71)
Primary location	Pheochromocytoma 6
	Paraganglioma 17
	Synchronous pheochromocytoma and paraganglioma 2
Multifocality	Multifocal or metastatic 17
	Unifocal 8
Genetic syndrome	Sporadic 8
	SDHB 4
	SDHD 9
	SDHA 2
	FH 2
Scan purpose	Staging-initial diagnosis 4
	Follow up 18
	Therapeutic decision 3
Follow up after scan	10 months (1–25)
Number of patients with lesions located in	Cervical 15
	Bone 6
	Nodal 5
	Mediastinal 5
	Abdominal extra adrenal 4
	Liver 3
	Lung 2
	Adrenal 1

**Table 2:** Characteristics of each patient included in the series

Patient	Gender	Age	Initial diagnosis	Multifocality	Syndrome	PET/CT lesions	PET/MRI lesions
1	M	48	Pheochromocytoma	Yes	Sporadic	16	16
2	F	71	Cervical paraganglioma	Yes	SDHB	4	5
3	F	38	Cervical paraganglioma	No	Sporadic	1	1
4	M	38	Retroperitoneal paraganglioma	Yes	FH	5	5
5	M	54	Cervical paraganglioma	Yes	SDHD	4	4
6	F	53	Cervical paraganglioma	Yes	SDHD	8	8
7	M	60	Sella Turcica paraganglioma	No	Sporadic	1	1
8	F	46	Pheochromocytoma	Yes	Sporadic	11	16
9	F	50	Cervical paraganglioma	Yes	SDHD	4	4
10	F	53	Pheochromocytoma	Yes	Sporadic	14	19
11	M	46	Pheochromocytoma and cervical paraganglioma	Yes	SDHD	2	2
12	M	58	Pheochromocytoma and cervical paraganglioma	Yes	SDHD	5	5
13	F	38	Mediastinal paraganglioma	No	SDHD	1	1
14	M	59	Cervical paraganglioma	Yes	SDHB	3	4
15	F	54	Cervical paraganglioma	Yes	SDHD	5	7
16	M	31	Retroperitoneal paraganglioma	Yes	SDHB	3	4
17	F	52	Cervical paraganglioma	No	Sporadic	1	1
18	F	63	Cervical paraganglioma	Yes	SDHA	3	3
19	M	44	Pheochromocytoma	No	Sporadic	1	1
20	M	51	Cervical paraganglioma	Yes	SDHD	7	8
21	M	20	Pheochromocytoma	No	SDHA	1	1
22	F	43	Retroperitoneal paraganglioma	Yes	FH	4	4
23	M	27	Cervical paraganglioma	No	SDHB	1	1
24	M	35	Pheochromocytoma	Yes	Sporadic	3	3
25	F	56	Cervical paraganglioma	No	SDHD	1	1

PET/CT detected 109 lesions compared to the 125 lesions detected on the PET/MRI, without any statistical significance (p: 0.721 Mann–Whitney U test). No statistical significance was obtained based on the distinct locations (liver p: 0.918, bone p: 0.748, cervical p: 0.959, abdominopelvic p: 0.988, lung p:1, nodes p: 0.979, mediastinum p: 0.989), and these results did not show significance in multifocal disease either. No significative differences were observed between the lesions detected by both imaging tests and multifocality or the genetic syndrome (least squares regression p: 0.566 and p: 0.683, respectively).

**Fig. 1:**
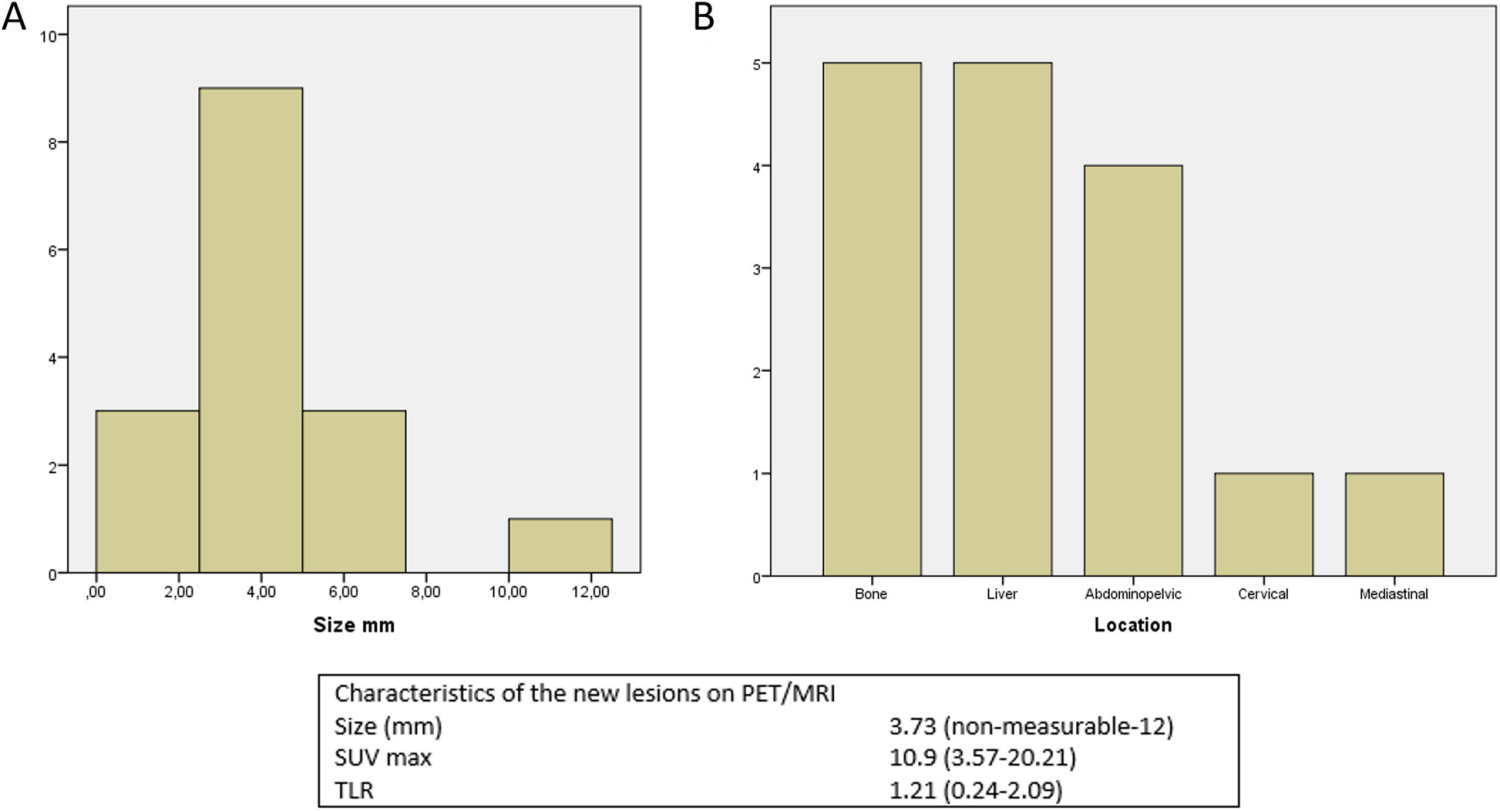
**A** and **B** Size and location distribution per number of new lesions detected on PET/MRI. Bottom panel: size and intensity of new lesions detected on PET/MRI

PET/MRI detected 16 new lesions (5 liver, 5 bone, 3 abdominopelvic, 1 lymph node, 1 mediastinal, 1 cervical), increasing the diagnostic accuracy of PET/MRI by 14.6% compared to PET/CT. The mean size of new lesions was 3.73 mm, SUVmax 10.9 (3.57–20.21) and mean TLR was 1.21 (0.24–2.09). All new lesions were detected in patients who had already known multifocal disease (Fig. 1).

**Fig. 2:**
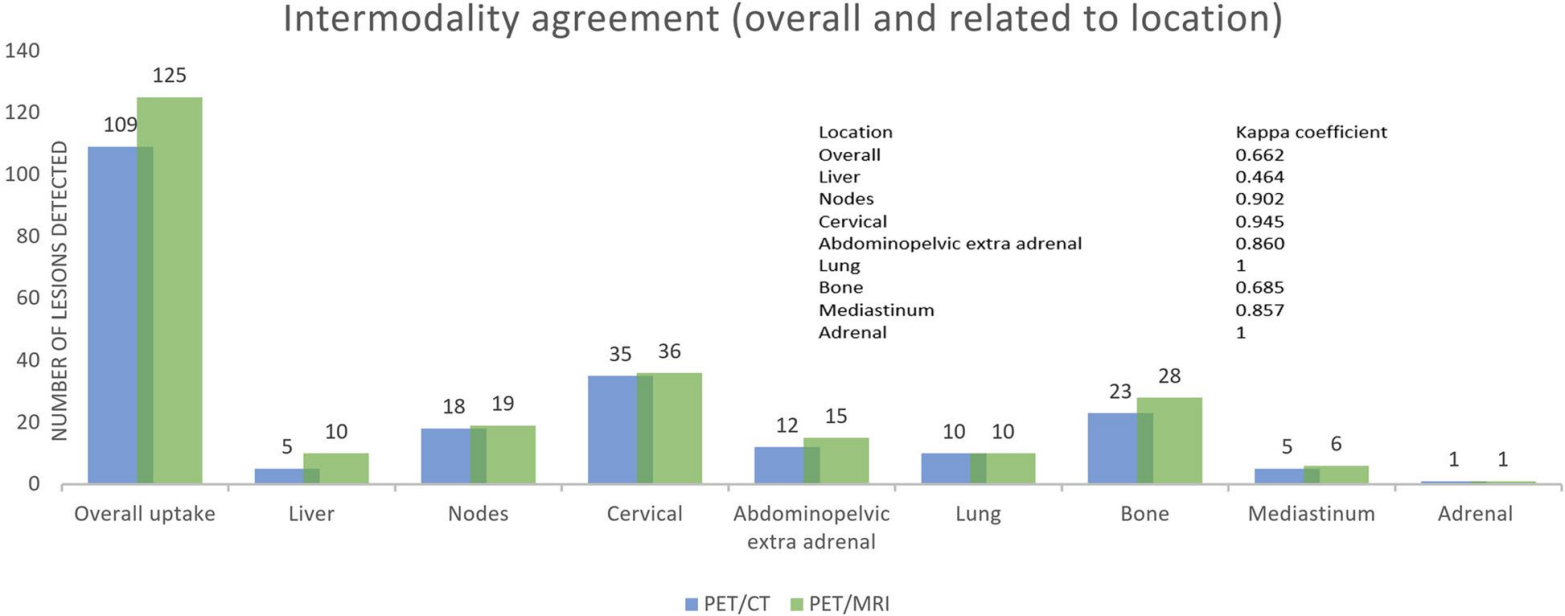
Intermodality agreement between PET/CT and PET/MRI, overall and by locations

There was good overall intermodality agreement between both scans (kappa coefficient 0.662). By locations, the best correlations were observed in the lymph nodes and the cervical location with an almost perfect agreement (Kappa coefficient 0.902 and 0.945), whereas the most discrepant locations were liver and bone (Kappa coefficient 0.464 and 0.685), with the hepatic location showing only moderate agreement. The intermodality agreement was slightly worse in both liver and bone if only multifocal disease was analyzed (Kappa coefficient 0.446 and 0.660 respectively) (Fig. 2).

On average PET/MRI obtained a higher SUVmax uptake and higher TLR per lesion than PET/CT (38.27 vs 19.45 and 4.12 vs 2.44 respectively) with statistically significant differences between them (p: 0.000 U Mann–Whitney test in TLR). The TLR difference between both scans per lesion and overall intrapatient were 1.67 and 2.30 in favour of PET/MRI. Tumor and normal tissue SUVmax and SUVmean are described in Table 3.

**Table 3:** Average lesions and reference tissues SUVmax, SUVmean, range and standard deviation

	**Mean**	**Range**	**Standard deviation**
PET/CT Lesion SUV max	19.45	0.90–202.18	33.17
PET/MRI Lesion SUV max	38.27	1.05–295.37	51.41
PET/CT Liver SUV max	8.72	4.84–14.23	2.57
PET/CT Liver SUV mean	6.47	3.18–10.39	1.85
PET/MRI Liver SUV max	10.51	5.40–18.05	3.24
PET/MRI Liver SUV mean	7.28	3.63–10.51	2.08
PET/CT Spleen SUV max	27.34	13.91–41.55	8.31
PET/CT Spleen SUV mean	22.20	10.66–36.86	7.41
PET/MRI Spleen SUV max	33.30	17.19–56.46	11.27
PET/MRI Spleen SUV mean	26.34	12.77–44.33	8.09

**Fig. 3:**
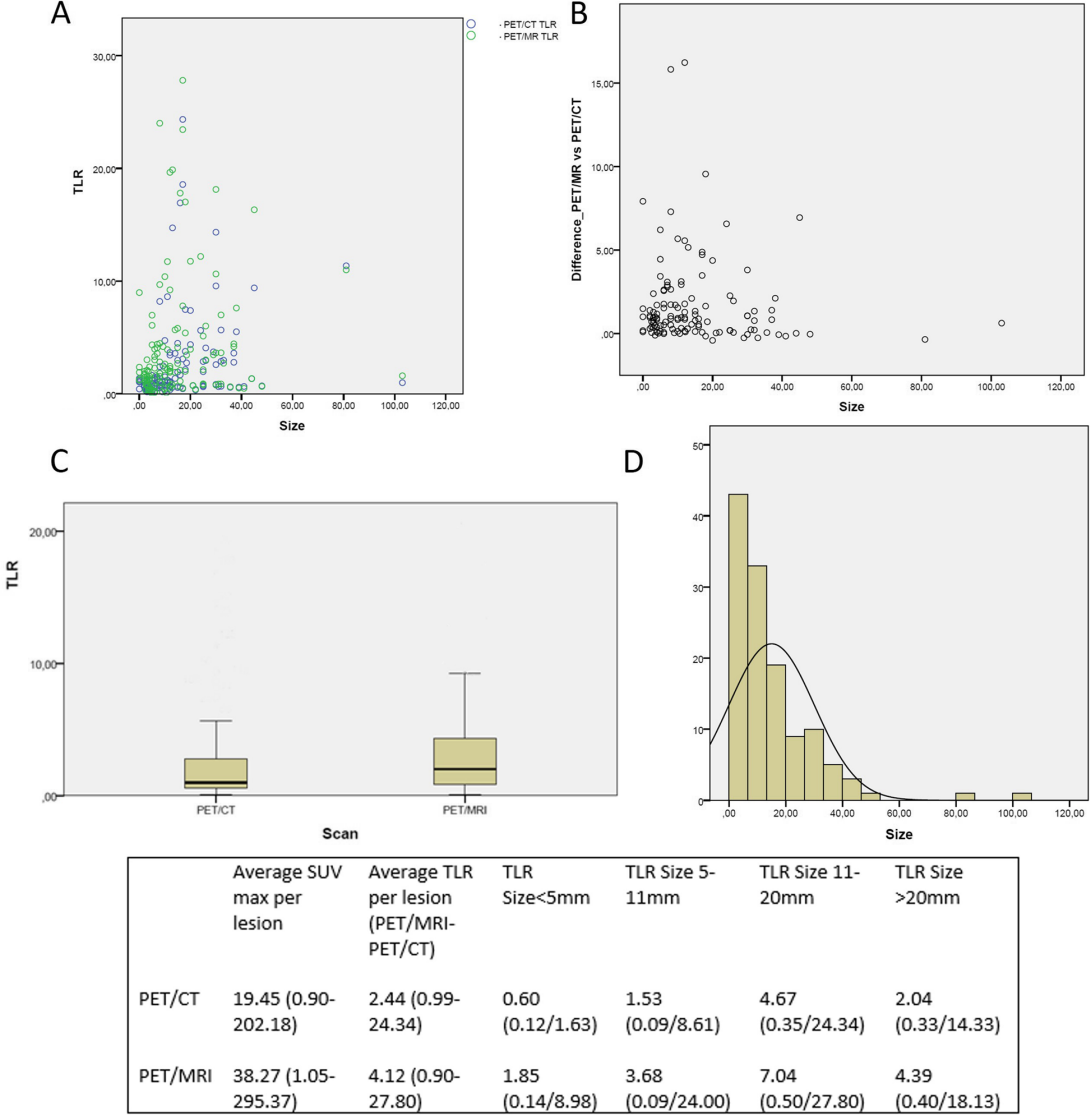
**A** TLR values of PET/CT and PET/MRI as a function of lesion size. Blue circles correspond to PET/CT and the green ones to PET/MRI. **B** TLR differences between PET/MRI and PET/CT as a function of lesion size. **C** Box plot of TLR in PET/CT and PET/MRI. **D** Size distribution of sample lesions. Bottom panel: Overall SUVmax and TLR values, as a function of size

**Fig. 4:**
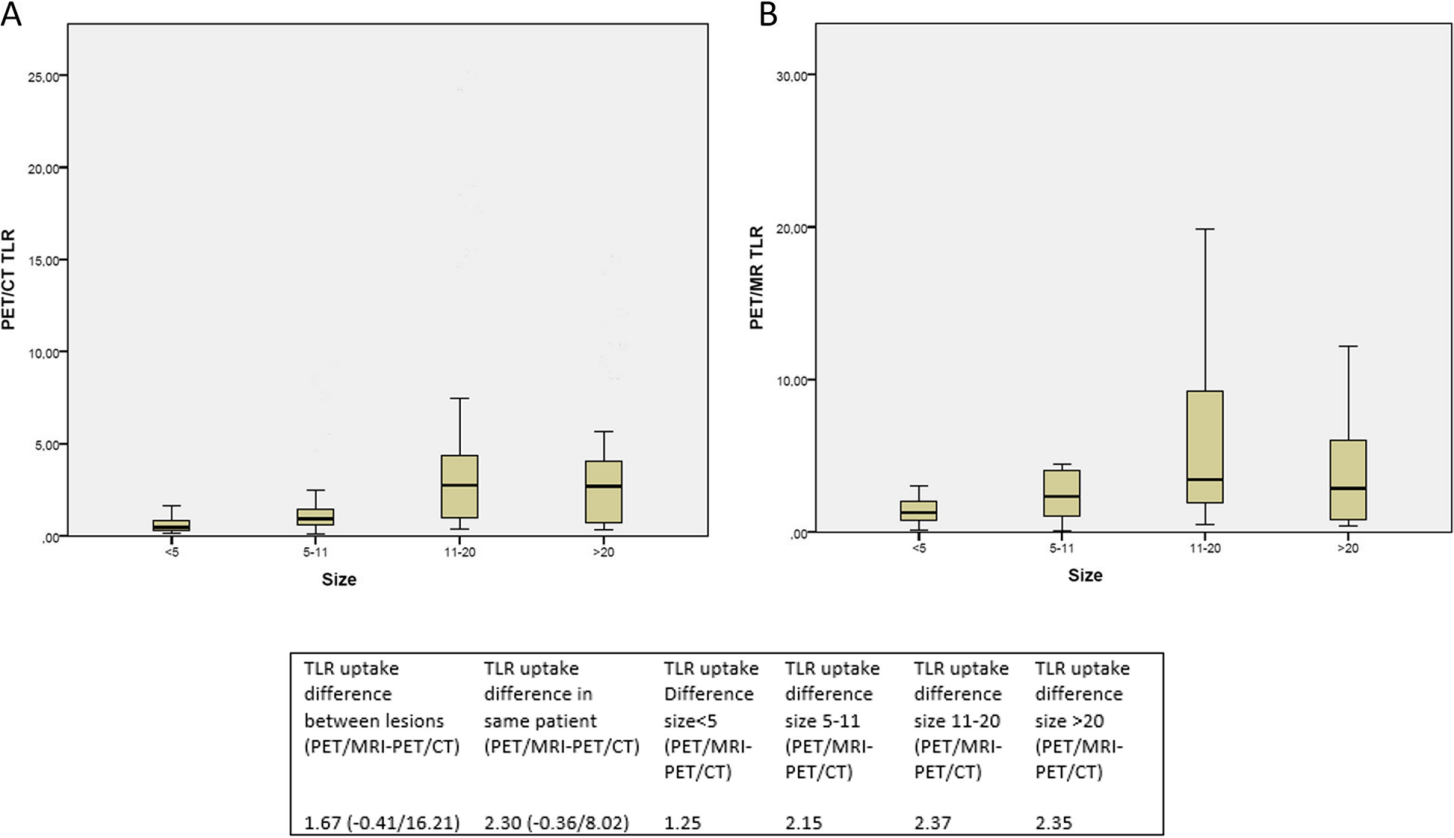
**A** Distribution of TLR values ​​in PET/CT according to quartiles of lesion size. **B** A. Distribution of TLR values ​​in PET/MRI according to quartiles of lesion size. Bottom panel: Difference in overall TLR, by patient and by size

Statistically significant differences were also observed in TLR between both scans related to the size groups (p: 0.023 Kruskall Wallis for independent samples). The greatest difference in uptake between PET/MRI and PET/CT was observed in the larger lesions (size 11–20: 2.37, size > 20: 2.35, size < 5 mm: 1.25 and size 5-11 mm: 2.15) (Figs. 3 and 4).

**Fig. 5:**
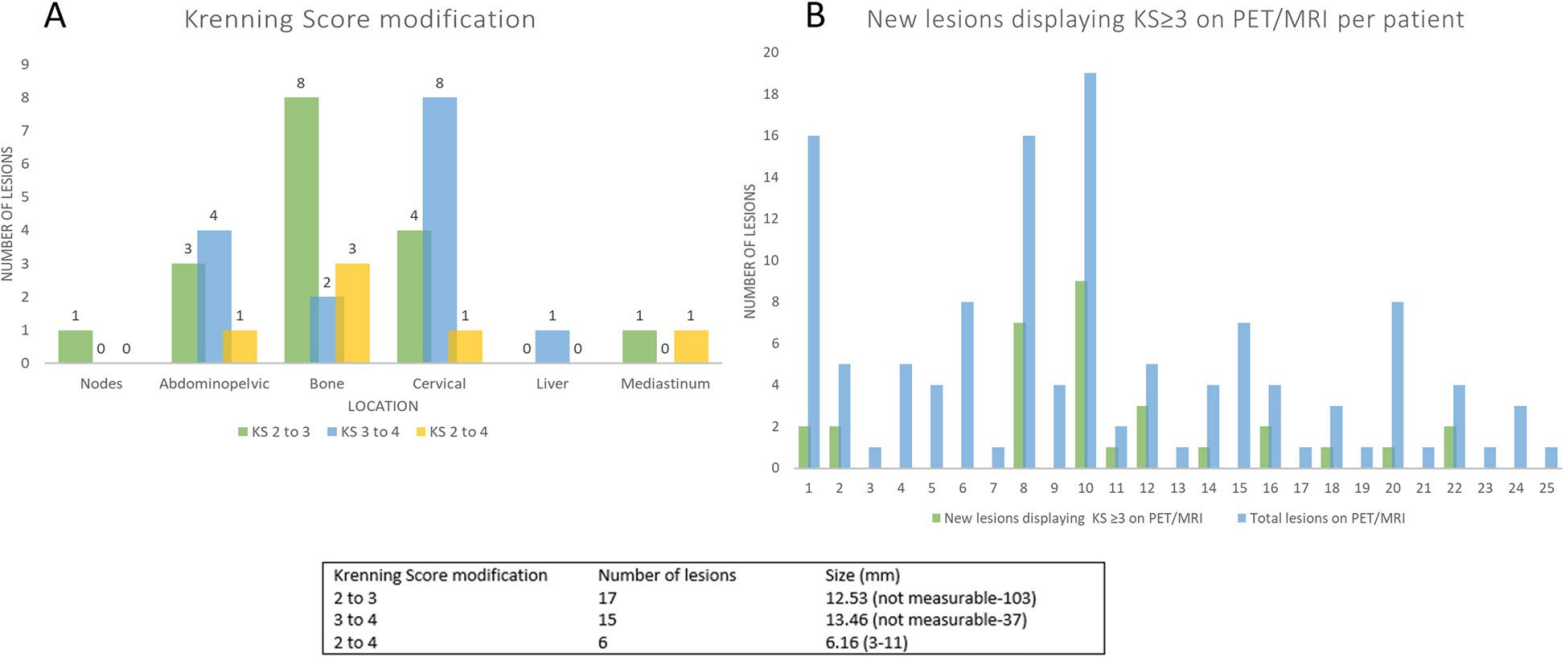
Histograms. **A** Distribution of the lesions that modified the Krenning Score by location. **B** New lesions displaying a Krenning Score ≥ 3 on PET/MRI, including pre-existing lesions on PET/CT that increased their uptake on PET/MRI, and newly detected lesions on PET/MRI with a Krenning Score ≥ 3. Bottom table: number and mean size of lesions according to the modification of the Krenning Score

Regarding the intensity of uptake according to the KS, a maximum score of 4 was obtained in 19 patients, a score of 3 in 4 patients and only one patient obtained a score of 2 and 1. In 15 of the 25 patients, an upward modification of the KS was observed in the PET/MRI, with modification in 38 of the 125 lesions studied. The mean size of lesions with a change in KS was 11.89 mm (not measurable-103 mm), with a mean SUVmax of 9.03 (2.03–23.78) in PET/CT and 30.85 (7.34–96.68) in PET/MRI. The overall mean TLR increased from 1.15 (0.28–3.60) in PET/CT to 3.24 (1.02–9.68) in PET/MRI. 17 lesions increased in PET/MRI the KS from 2 to 3, 15 lesions from 3 to 4, and 6 lesions changed from KS from 2 to 4. When adding the lesions that increased the KS, to the new lesions detected with higher uptake than the liver, a total of 31 more lesions displayed a KS ≥ 3 on PET/MRI compared to PET/CT. Per patient, in the overall series a mean of 16.97% of lesions displayed a KS ≥ 3 on PET/MRI, that were not detected or showed lower KS on PET/CT. If only patients with multifocal disease were assessed, this mean rose to 24.96% per patient. The distribution of KS changes by location, size and per patient are summarized in Fig. 5.

**Fig. 6:**
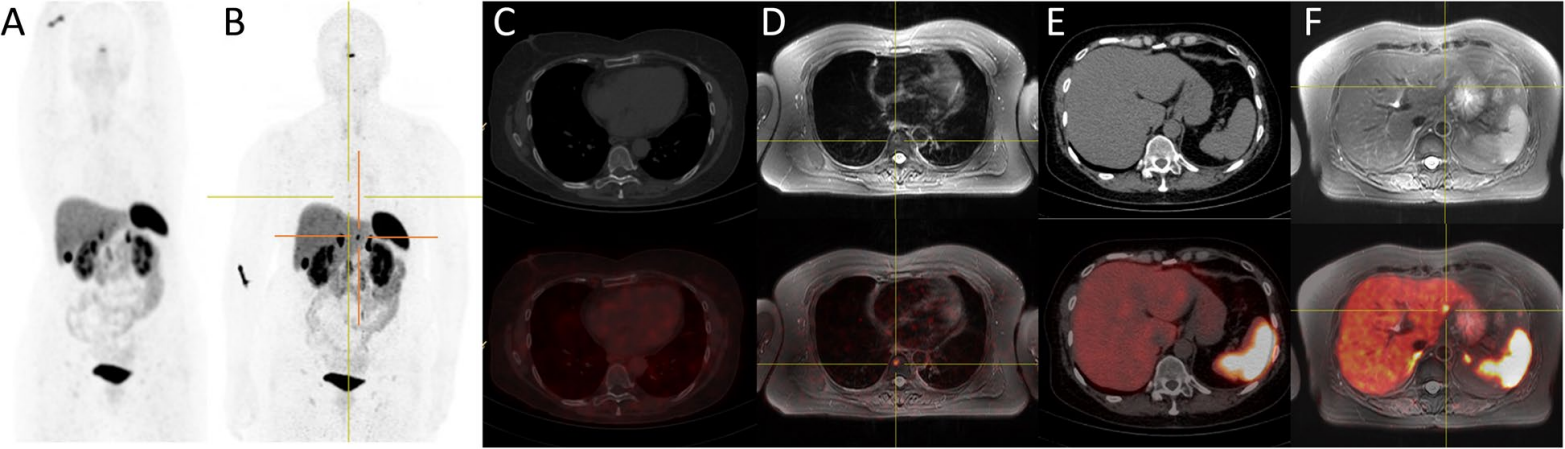
Comparative images between PET/CT and PET/MRI in patient number 15. PET/MRI revealed a new bone lesion (involvement of a new organ) and a new hepatic lesion (organ already affected). **A** Maximum intensity projection (MIP) in PET/CT. **B** MIP in PET/MRI. **C** PET/CT in bone reconstruction. **D** PET/MRI in FS T2-weighted sequence. A new lesion is detected in the T8 soma showing a central signal hypointensity and a peripheric hyperintensity signal, with intense uptake on PET. **E** PET/CT in soft tissue reconstruction. **F** PET/MRI in FS T2-weighted sequence. A new hepatic lesion is observed in segment II, hyperintense in FS T2- weighted sequence and with intense uptake in PET. This uptake was difficult to discriminate on PET/CT from hepatic baseline heterogeneous activity

In 8 patients, all of them already metastatic, there was a mismatch between PET/CT and PET/MRI, due to the appearance of new lesions. In one of these patients, it was due to the involvement of a new organ (patient 15, with a new bone lesion), and in the remaining 7 patients due to new lesions in organs already affected (Fig. 6).

In 3 of the 25 patients (12%), PET/MRI modified the clinical management. Among the 3 patients referred for therapeutic decision, in those 2 with metastatic disease (patients 1 and 11), PET/MRI supported SSR-targeted therapy. In patient 1, two lesions increased the KS above 3, but the main contribution of the PET/MRI was to confirm, thanks to the MRI sequences, that a splenic lesion was not viable but rather an area of ​​necrosis. By confirming this lesion as a true negative, SSR-therapy could be planned ruling out that no lesions were left untreated. In patient number 11, displaying two lesions, PET/MRI morphologically confirmed one of them as a left tympanic PGL and changed its KS from 2 to 3. On the other hand, in patient 24 referred for follow-up, PET/MRI ruled out disease over millimetric bone morphological lesions, so active surveillance was decided. In the rest of the patients referred for follow-up and staging, the contributions of PET/MRI did not justify a change in the clinician's management.

## Discussion

It is difficult to compare the diagnostic accuracy of [^68^ Ga]Ga-DOTA-TOC PET/CT and PET/MRI due to the high sensitivity and specificity of the radiopharmaceutical by itself [13].

[^68^ Ga]Ga-DOTA-TOC PET/MRI, has certain advantages over conventional PET/CT. High-energy positron emitters such as ^68^ Ga are especially suitable for PET/MRI. This isotope has a shorter range before its annihilation due to the influence of the magnetic field of MRI, improving the spatial resolution compared to PET/CT [14]. In contrast to PET/CT, in PET/MRI the acquisition of both images is simultaneous. This fact allows for an increase in the acquisition time of PET in PET/MRI, reduces noise, improves the detection sensitivity in small lesions, and avoids artifacts in co-registration caused by respiratory movements, particularly in the upper abdominal organs.

In addition to the superior soft-tissue contrast due to MRI, [^68^ Ga]Ga-DOTA-TOC PET/MRI offers double functional information, SSR expression by PET acquisition, and water diffusion if the DWI is acquired [15].

The incorporation to the new PET/MRI equipment of the combination of TOF, which reduces image noise and improves contrast, and PSF, which locates the annihilation points along the line-of-response at the true geometric locations, improves the detection of small lesions considerably [16, 17].

Thanks to the information provided by PET, the improved TOF and the PFS, fewer MRI sequences are necessary in PET/MRI, therefore shortening the exploration time. Nevertheless, the examinations are still significantly longer and more uncomfortable than those of PET/CT [18]. This fact, added to the few PET/MRI pieces of equipment available, and their high cost, are its main drawbacks.

The mean activity of [^68^ Ga]Ga-DOTA-TOC corrected by radioactive decay at the start of PET/MRI acquisition was 36.37% lower than the radioactivity at the start of PET/CT. Despite the lower proportional activity of [^68^ Ga]Ga-DOTA-TOC in PET/MRI, it detected 14.6% more lesions than PET/CT, meaning that the radiation to be administered in PET/MRI could be adjusted downwards in the future, without affecting its diagnostic performance. Also, the absence of ionizing radiation from CT makes PET/MRI especially feasible in young patients who will require lifelong follow-up, due to active disease or because they are carriers of a genetic syndrome, although the reduction of accumulated radiation is a benefit whose real future impact is still unknown.

The most discrepant locations between PET/CT and PET/MRI were liver and bone. Both in the liver and in the bone, PET/MRI detected 5 more lesions than PET/CT, followed by abdominopelvic lesions (3 lesions), with a millimetric size (mean size 3.73 mm) and always in patients with known metastatic disease. In addition to the technical improvements of PET, these differences are probably due to the higher capacity of MRI in the detection of hepatic liver and bone marrow small lesions [19]. Apart from location, size was a key factor in the detection of new lesions on PET/MRI. With a size ranging between uptakes impossible to characterize morphologically to lesions of maximum 12 mm, the new lesions were below the spatial resolution of PET/CT.

Both SUVmax and TLR were higher on PET/MRI than on PET/CT (38.27 vs 19.45 and 4.12 vs 2.44 respectively). Coura-Filho et al., evaluated the [^68^ Ga]Ga-DOTA-TATE temporal variation of SUVmax in normal tissues and NETs. They acquired studies before 15 min, between 45–90 min and after 90 min post-injection, concluding that there was stability in SUVmax values over time both in tumors and normal tissues [20]. This study, in addition to the fact that our TLR values also increased proportionally to the SUV, minimizes the possibility that the higher uptake in PET/MRI is due to physiological changes in the biodistribution of the radiopharmaceutical, since the PET/MRI is a later study.

The most pronounced increases in uptake intensity were observed in larger lesions (size 11-20 mm and > 20 mm). However, in these sizes, this variation had no clinical impact, as both PET/CT and PET/MRI showed high uptake intensity (TLR in size 11-20 mm changed from 4.67 to 7.04 from PET/CT to PET/MRI and in size > 20 mm from 2.04 to 4.39). It is in the smallest lesions where, this difference had more diagnostic impact, despite a smaller variation in the uptake intensity between both scanners. For < 5 mm lesions, the mean TLR increased from 0.6 on PET/CT to 1.85 on PET/MRI. This means that the lesions changed from having notably lower uptake than baseline hepatic uptake (on PET/CT), to having intense uptake equal or greater than hepatic uptake on PET/MRI, leading to a change in KS and getting the lesions over the line for suitability of PRRT.

The finding of these changes in the smaller lesions had an important impact on the modifications of the KS. KS was introduced into the functional SSR imaging of NETs, with the aim of semi-quantitatively grading the pathological uptake of lesions. A KS with a value of 0 means no uptake; KS1 very low uptake; KS2 uptake less than or similar to the liver; KS3 uptake greater than liver; KS4 uptake greater than the spleen [21]. This score was initially introduced to assess planar octreotide scintigraphy and was later adapted for PET with [^68^ Ga] Ga-DOTA-related radiopharmaceuticals. A KS ≥ 3 has important clinical implications. The high expression of SSRs in the functional image means that there is a therapeutic target for “cold” somatostatin analogs or PRRT. In PRRT, the reports recommend a KS ≥ 3 for effective therapy [22]. In our series, the PET/MRI implied an upward elevation of the KS. In up to 60% of the patients, the KS changed in some of the lesions. 30.4% of the evaluated lesions experienced a change in the KS, 18.4% of the lesions increased their KS from 2 on PET/CT to a KS ≥ 3 on PET/MRI and most importantly, a 24.96% of the lesions per patient with multifocal disease displayed a KS ≥ 3 on PET/MRI, that were not detected or showed lower KS on PET/CT. From a theragnostic point of view, these lesions would change from not candidates for SSR-targeted therapies to being suitable for these treatments.

The mean size of the lesion’s changing KS was 11.89 mm. This result implies a reduction in the size of the lesions that can be characterized by [^68^ Ga]Ga-DOTA-TOC PET/MRI compared to previous studies with PET/CT. Hope et al. described that the change from using [^111^In]In-octreotide scintigraphy to [^68^ Ga]Ga-DOTA-TATE PET/CT led to an increase in KS, particularly in lesions smaller than 2 cm. In our study, the change from conventional [^68^ Ga]Ga-DOTA-TOC PET/CT to PET/MRI technology supposes an increase in the KS of lesions of approximately 1 cm [23].

In 3 of the 25 patients, we consider that PET/MRI modified the clinical management (12% of patients). This result is highly influenced by the reason for patient referral to the scan. 22 of the 25 patients were referred for staging or follow-up. In the context of follow-up, the appearance of a millimetric lesion, or the increase in uptake intensity, was considered as a consequence of a better resolution of the PET/MRI and not as a real progression of the disease. In the context of staging, all the patients in whom there was a mismatch were already metastatic patients, so they already were referred to systemic treatment if required.

The major changes occurred in patients referred for therapeutic decision (changes applied to 2 out of 3 patients), but this was the smallest group of patients. Considering the changes in the KS in our overall series, if this group had been more numerous, we believe PET/MRI would have probably modified the strategy, or at least reinforced the therapeutic decision in more cases.

The third case in which PET/MRI had a clinical impact was patient 24. This patient with a metastatic PHEO, previously received two [^131^I]MIBG treatments, with persistence of sclerotic bone lesions of a few millimeters, below spatial resolution capacity of PET/CT. In this case, PET/MRI, which in our series has shown great accuracy in quantifying small lesions, confirmed a very low uptake and therefore active disease, so a follow-up was decided.

To our knowledge, no similar series have been published on PGGLs. There are few series comparing in a same-day protocol PET/CT and PET/MRI with [^68^ Ga]Ga-DOTA related radiopharmaceuticals in NETs, but in most of them, the intensity of uptake is not assessed. Berzaczy et al. published a series comparing [^68^ Ga]Ga-DOTA-NOC PET/CT and subsequent PET/MRI on the same day in 28 patients, but the study included patients with NETs (with different degrees of differentiation) of small bowel, lung, pancreas, colon, unknown primary location and two PGLs. Berzaczy et al. observed comparable results between PET/CT and PET/MRI with a minor advantage for PET/MRI in terms of overall accuracy and sensitivity. However, in their study Berzaczy et al., observed differences in the SUV between both techniques, but with higher values ​​measured on PET/CT than on PET/MRI, unlike in our series. These results do not fit the evolution of uptake in normal and tumor tissues that has been described previously, and it is probably due to the characteristics of the scanners used. The PET/CT equipment performed in this study incorporated TOF while the PET/MRI did not. Differences between both systems result in overall better image quality with higher background activity and small uptake volumes in the equipment using TOF [24].

Savicki et al. compared PET/CT and PET/MRI with [^68^ Ga]Ga-DOTA-TOC in 30 patients with proven NETs, demonstrating that out of 70 liver lesions, 10 were not detected by PET/CT, and that all of them measured < 1 cm. These results are similar to those of our protocol [25].

Seith et al. compared retrospectively in 29 patients, fast non‑enhanced abdominal examination protocols in PET/MRI to PET/CT in NETs. They concluded that non-enhanced contrast PET/MRI is an alternative for follow-up examinations in patients with unresectable NET and kidney failure [26].

Beiderwellen et al. studied the usefulness of PET/MRI with [^68^ Ga]Ga-DOTA-TOC in gastroenteropancreatic NETs in 8 patients, with similar results between both techniques, observing high soft tissue contrast in MRI and special utility in patients with chronic renal failure with whom iodinated contrast could not be used [27]. Hope et al. reported a simultaneous study with [^68^ Ga]Ga-DOTA-TOC in 10 patients with known liver metastases, with better liver disease detection in PET/MRI, just as in our study, but with improved detection of nodal involvement in PET/CT [28].

In economic terms, Mayerhoefer et al. performed a cost comparison between PET/CT and PET/MRI, in a mixed oncologic patient population, using two measures of effectiveness: the percentage of diagnostic accuracy and changes in clinical management. They observed that PET/MRI led to changes in clinical management or therapy in 8% of patients (+ 13.4% accuracy compared with PET/CT). These results included NETs due to the superior detection of liver metastases and are of a similar range as our results (12% of changes in clinical management). The cost per PET/MRI examination was nearly 50% higher compared with that of PET/CT, but it must be contrasted with the higher diagnostic accuracy of PET/MRI and the ability to affect management and therapy decisions in a significant fraction of patients. The incremental cost-effectiveness ratios of PET/MRI were 14.26 EUR per percent of diagnostic accuracy, and it was 23.88 EUR per percent of correctly managed patient. They concluded that a histology-based triage of patients to either PET/MRI or PET/CT could be meaningful, being one of the reasons that promoted our study [29].

But in addition to the cost, accessibility to PET/MRI should be assessed, considering that it is not available in all medical centers and that it is still a longer and more uncomfortable examination. Therefore, it is necessary to determine which patients will benefit from PET/MRI versus PET/CT, especially when only a 13.4% difference in accuracy has been described between both techniques.

**Fig. 7:**
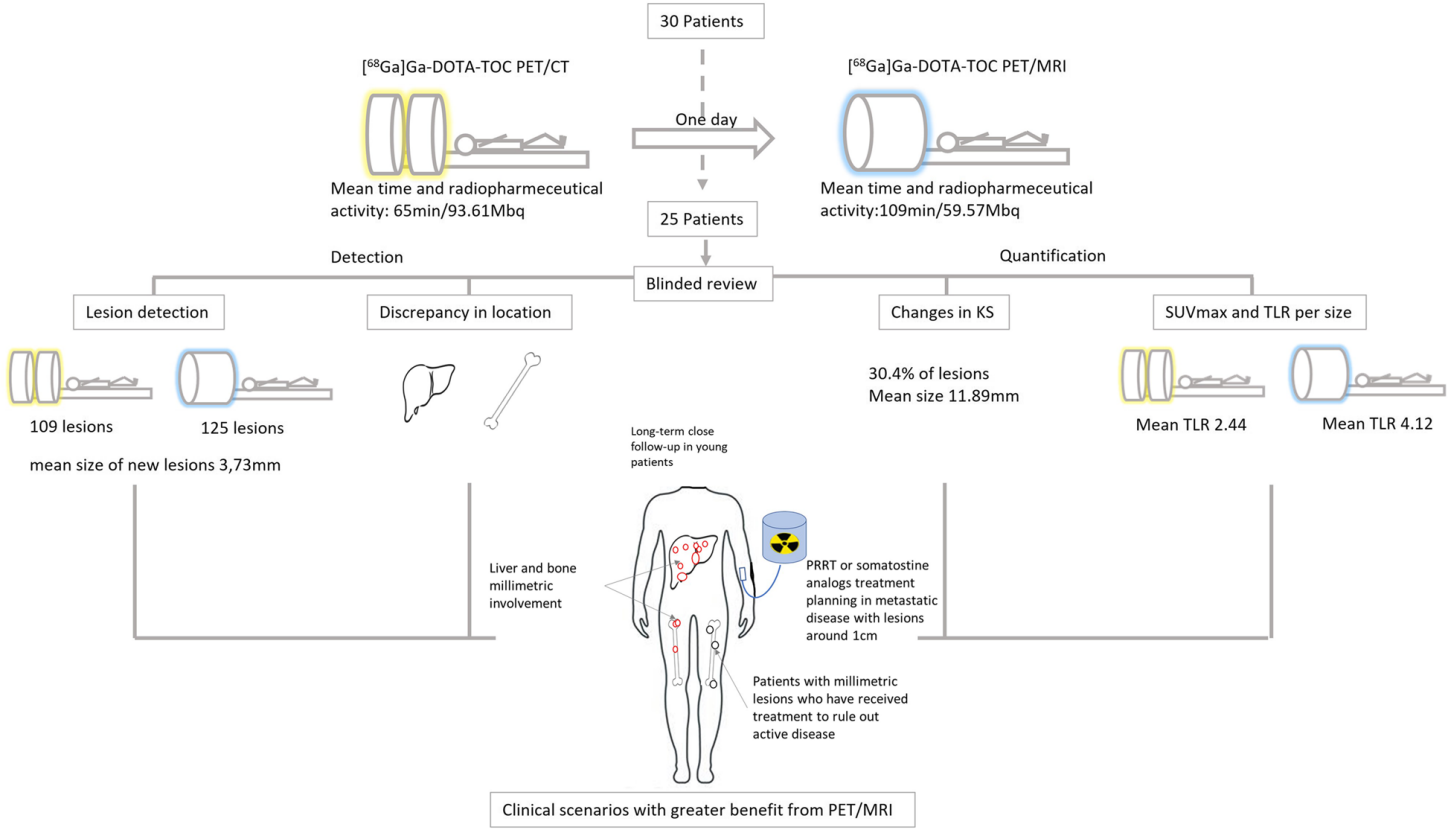
Diagram of the study and the profiles of patients with probable greater benefit from performing PET/MRI

After evaluating our results, based on the lower proportional activity of the radiopharmaceutical used in PET-MRI, the greater discrepancy in certain locations between both scans, the size of the lesions that showed change in quantification and the reason for referral of the patient, the clinical scenarios that could increase the benefit of PET/MRI over PET/CT would be in our opinion (Fig. 7):

- Young patients, with active disease or carriers of a genetic syndrome, with good tolerance to the MRI examination, which require lifelong surveillance, in whom the ionizing radiation of the CT would be avoided, and the activity of the administered radiopharmaceutical could probably be reduced.
- Patients in follow-up with liver and bone predominance of small-sized metastatic disease (a few millimetres).
- Patients undergoing evaluation prior to SSRs-targeted treatment (somatostatin analogs or PRRT), with the presence of small-sized metastatic disease (around 1 cm).
- Patients with millimetric metastatic disease who have received systemic therapy, and in whom PET/CT cannot determine the presence of active disease.

Within the limitations of the study, the reference standard was based mostly on clinical follow-up and imaging follow-up, and to a lesser extent on the histopathology of surgical specimens and biopsies. Intravenous contrast agents were not used in either PET/CT or PET/MRI. This was solved by using [^68^ Ga]Ga-DOTA-TOC, which by itself has high accuracy, and can provide information as if it were a contrast agent, in addition to the DWI sequence as described above. All patients underwent PET/CT first and PET/MRI next, which could constitute a bias in uptake quantification. Technological improvements of PET/MRI over PET/CT may have increased the differences in the quantification of lesions. The detection of small lung nodules has traditionally been one of the weaknesses of MRI. However, lung involvement is rare in PGGLs (in our series, 2 out of 25 patients), our patients underwent respiratory monitoring to motion correction, and PET uptake of the lesions facilitated their location, so we did not observe differences between the two tests in this location [30]. Finally, the patients were referred for examination for varied reasons, so their change in clinical management was more difficult to assess. However, given the homogeneity of our series, we believe that the results can be extrapolated to the separate groups, as described in Table 1, according to the reason for referral.

## Conclusion

The progressive improvement in nuclear medicine equipment, and its association with MRI, is allowing the detection of more small lesions with significant SSR expression which were only faintly SSR expressive or invisible on PET/CT. PET/MRI showed minor advantages over conventional PET/CT in the detection of new lesions but increased the intensity of SSRs expression in a substantial number of them.

In the current environment of personalized medicine and theranostics, these findings support careful patient selection for PET/MRI to minimise lifetime cumulative radiation dose, underdiagnosis and better quantification of SSR expression for targeted therapy. Whether the latter would translate to an improved patient outcome is unknown.

## Data Availability

The datasets used and/or analysed during the current study are available from the corresponding author on reasonable request.

## Abbreviations

PET: Positron emission tomography
MRI: Magnetic resonance imaging
CT: Computed tomography
PGLs: Paragangliomas
PHEOs: Pheochromocytomas
PGGLs: Paragangliomas and pheochromocytomas
KS: Krenning Score
SUV: Standard Uptake Value
TLR: Target-to-liver-ratio
NETs: Neuroendocrine tumors
SSRs: Somatostatin receptors
PRRT: Peptide Receptor Radionuclide Therapy
FOV: Field of view
TOF: Time-of-flight
PSF: Point-Spread Function
FS: Fat-suppressed T2-weighted sequences
DWI: Diffusion-weighted sequences
